# Growth history revealed by tree rings provides clues for the conservation of an endangered subtropical tree species

**DOI:** 10.3389/fpls.2026.1868379

**Published:** 2026-06-16

**Authors:** Hengfeng Jia, Yanbing Yang, Xiangwen Hou, Haibo Li, Qifang Li, Chongyi Yang, Xufen He, Yunli Jiang, Zaiqi Luo, Hongyan Qiu

**Affiliations:** 1 Guizhou Academy of Forestry, Guiyang, Guizhou, China; 2 Guizhou Fanjingshan Forest Ecosystem Observation and Research Station, Jiangkou, Guizhou, China; 3 Administration Bureau of Fanjing Mountain National Nature Reserve, Jiangkou, Guizhou, China; 4 Guizhou University, Guiyang, Guizhou, China; 5 Meteorological Bureau of Dejiang County, Dejiang, Guizhou, China; 6 State Key Laboratory of Vegetation and Environmental Change, Institute of Botany, Chinese Academy of Sciences, Beijing, China; 7 China National Botanical Garden, Beijing, China

**Keywords:** *Abies fanjingshanensis*, disturbance history, growth decline, growth release, tree-ring

## Abstract

**Introduction:**

*Abies fanjingshanensis* is an endangered relic conifer endemic to Fanjing Mountain in Guizhou Province, Southwest China. Its sparse population, limited regenerative capacity, and fragile habitat underscore the urgent need for evidence-based conservation strategies.

**Methods:**

In this study, dendrochronological methods were applied to quantify radial growth dynamics, identify climatic constraints, and reconstruct disturbance history.

**Results:**

The mean radial growth rate of *Abies fanjingshanensis* was 1.439 mm yr-1. Tree growth exhibited a significant negative correlation with June temperature and a positive correlation with the drought index (scPDSI) from July to September (*p* < 0.05), indicating that both high summer temperature and water deficit constrain growth, with moisture availability as the dominant limiting factor during the growing season. Disturbance reconstruction revealed three major events, a pronounced growth release in the early 1960s, likely triggered by widespread canopy mortality following the extreme drought of the late 1950s, and two growth suppression periods occurred in the early 1970s and around the turn of the 20th century.

**Discussion:**

Notably, radial growth has declined significantly over the past decade, accompanied by increased tree mortality, suggesting an emerging vulnerability of this species to ongoing climatic warming and drying. These findings highlight the critical role of hydroclimatic stress in regulating growth and survival of *Abies fanjingshanensis*. Reconstructing its growth and disturbance history provides key insights into population health and resilience, and offers a robust scientific basis for developing targeted conservation and management strategies under future climate change.

## 1 Introduction

The rapid loss of biodiversity has become one of the most pressing ecological crises globally. As a vital component of biodiversity, rare and endangered plants not only sustain the stability of forest ecosystems but also possess invaluable genetic resources (Huang et al., 2023; Asatulloev et al., 2025; Zhao et al., 2025). Nevertheless, many rare plants face a high risk of extinction due to a combination of intrinsic limitations (e.g., slow growth, poor regeneration) and external pressures such as human activities, drought, hurricanes, and pest outbreaks (University of Massachusetts Amherst, 2019; Zhang et al., 2026). Consequently, understanding population status, regeneration patterns, and the causes of endangerment has become a central goal in conservation biology.

Endemic to Mount Fanjing in Guizhou Province, China, *Abies fanjingshanensis* is an endangered relict plant and a nationally protected species (Huang, 2001; Wei et al., 2014; Xu et al., 2022). It plays an irreplaceable role in maintaining montane ecosystem stability, conserving water resources, and preserving biodiversity. Unfortunately, its natural regeneration is widely hindered, and its population continues to decline. The contributing factors include climate warming (which shrinks alpine habitats), a naturally narrow distribution range, and increasing human disturbance (Li et al., 2011; Mou et al., 2020). Previous studies have conducted basic surveys on its distribution and community characteristics, but systematic quantitative research remains lacking.

Three key questions remain unanswered. First, which climatic factors limit the radial growth of *Abies fanjingshanensis*? Second, how has its growth responded to historical disturbances? Third, have recent climate trends caused a persistent growth decline? To address these questions, we used dendrochronological methods with three specific objectives, (1) identify the main climatic factors controlling radial growth; (2) reconstruct historical growth dynamics and detect past growth release and suppression events; and (3) assess recent growth trends in the context of ongoing climate change. The results will provide a scientific basis for understanding the vulnerability of this species and for designing effective conservation strategies.

## 2 Materials and methods

### 2.1 Study area

We conducted this study on Mount Fanjing (27.83°~28.03°N, 108.77°~108.81°E), located at the junction of Jiangkou, Yinjiang, and Songtao counties in Tongren City, Guizhou Province, covering a total area of 775.14 km². The region has a subtropical humid monsoon climate, characterized by temperate conditions and ample sunshine. Annual mean temperature is from 13.1 to 14.7 °C, and total annual precipitation ranges from 1100 to 2600 mm, mostly falling from May to October. Relative humidity can reach 80%. Mount Fanjing features a well-defined vertical zonation of vegetation belts over an elevation difference of 2000 m (Gao et al., 2019; Yu et al., 2023; Luo et al., 2024).

### 2.2 Tree−ring data

**Figure 1:**
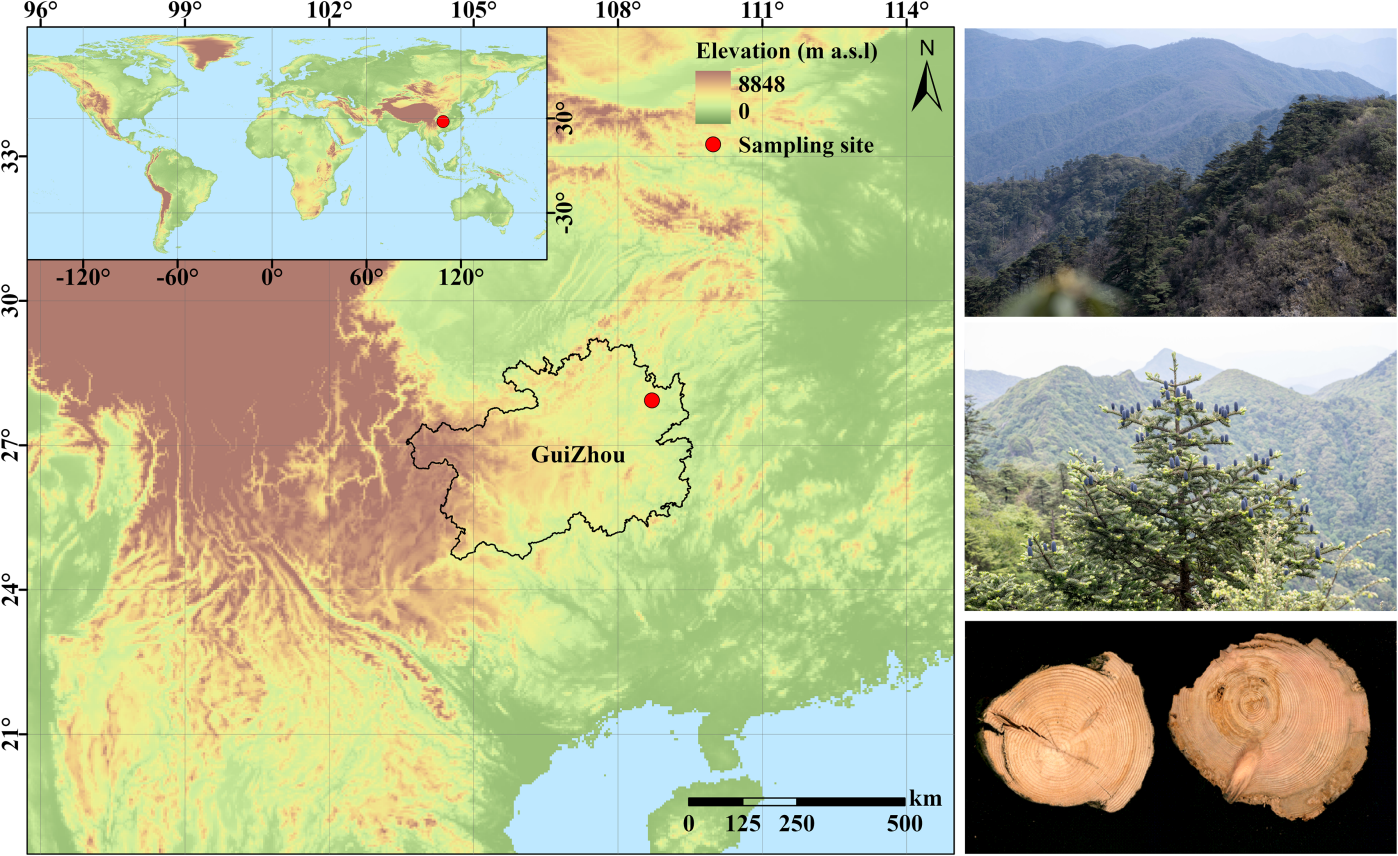
The location map of the sampling sites.

*Abies fanjingshanensis* is an endemic tree species of Guizhou Province, China, naturally distributed only on the upper northern slope of the Fanjingshan, where it exhibits a concentrated distribution with only a few scattered individuals. We conducted a field survey of its forest on the upper northern slope of Mount Fanjing from 8 to 12 November 2024. Six plots measuring 20 m × 20 m were randomly established in the concentrated distribution area. Within each plot, all individual trees were surveyed. *Abies fanjingshanensis* is a National Grade I protected plant in China, and sampling live trees is strictly prohibited. Due to the strict protection status, no living trees were sampled. Basal discs were collected from all fallen or dead *Abies fanjingshanensis* trees across its entire distribution range (**Figure 1**) that had intact basal sections, regardless of size or decay stage, provided the disc was sufficiently preserved for ring measurement. A total of 16 disc samples were collected, and for each sample, the identification number, diameter at breast height, and other relevant information were recorded (**Figure 1**).

In the laboratory, all samples were air-dried and then polished with successively finer sandpaper until the tree-ring boundaries were clearly visible under a microscope (**Figure 1**). The width of each tree ring was measured to an accuracy of 0.001 mm using the Lintab 6.0 system (Frank Rinntech Company, Heidelberg, Germany). Cross-dating of all samples followed standard dendrochronological techniques, ensuring that every ring was assigned to the calendar year in which it formed, the quality of cross-dating was assessed with the COFECHA program (Holmes, 1983; Fritts, 1999). To remove low-frequency growth trends related to the aging of each tree-ring sequence, a cubic smoothing spline with a 50% frequency-response cutoff was used (Cook, 1985, Cook et al., 1990). Finally, standard ring-width chronologies were generated using the ARSTAN program.

### 2.3 Competition relationships

The important value (IV) represents the status and role of a species within a community. IV = (Relative density + Relative frequency + Relative prominence)/3 for the canopy layer, and IV = (Relative density + Relative frequency + Relative coverage)/3 for the subcanopy and herbaceous layers. Niche breadth (B_L_) measures the degree to which a species utilizes resources (e.g., light, water, space, nutrients) or occupies environmental gradients (e.g., altitude, humidity, soil pH), reflecting its environmental adaptability and competitive ability for resources, which via the formula $B_{L}=1/\sum_{j=1}^{r}IV_{ij}$, where IV_ij_ denotes the proportion of the importance value of species i to the sum of all importance values on resource j and r is the number of quadrats. A larger niche breadth indicates a generalist species capable of exploiting diverse resources or thriving under varied environmental conditions, whereas a smaller niche breadth indicates a specialist species with strong dependence on specific resources or environments.

### 2.4 Analysis of climate-growth relationships

The monthly mean temperature, monthly total precipitation, and monthly scPDSI (self-calibrated Palmer Drought Severity Index) were sourced from https://climexp.knmi.nl/, where the climate data have a spatial resolution of 0.5° × 0.5°. For the sampling site, the climate conditions were calculated as the average of the data from the four closest grid points. Correlation analysis was performed to examine the relationships between climatic factors and tree growth. All analyses were conducted in R version 4.2.2 programs (R Core Team, 2022). The climatic variables included monthly mean temperature, monthly scPDSI, and monthly total precipitation, spanning from October of the previous year to September of the current year over the period 1963-2022. All plots were completed in Origin 2024.

### 2.5 Process modeling of tree radial growth

The Vaganov-Shashkin-Lite (VS-Lite) model is a reduced-form version of the VS model that simulates tree physiological processes using only monthly mean temperature, monthly total precipitation, and the latitude of the sampling site (Shashkin and Vaganov, 1993; Tolwinski-Ward et al., 2011, Tolwinski-Ward et al., 2013). In this study, the VS-Lite model was applied to investigate the climatic factors limiting the radial growth of *Abies fanjingshanensis*. The minimum values of the temperature−dependent growth curve (gT) and the soil moisture−dependent growth curve (gM) generated by the model reveal the growth−limiting factors at the study site for each year. During the growing season, tree growth is limited by monthly mean temperature when gT falls below gM, whereas growth is limited by soil moisture when gM falls below gT.

### 2.6 Growth trends and disturbances recorded in tree rings

The growth history of *Abies fanjingshanensis* was analyzed using the Mann-Kendall test (MK test) and the growth change percentage method (GC%). The MK test is a non−parametric statistical approach for detecting change points in time series (Liu, 2023). The GC% method evaluates growth trends using the formula GC% = (M_2_ - M_1_)/M_1_ × 100%, where GC% denotes the growth change percentage, and M_1_ and M_2_ represent the mean tree−ring index values for the preceding 10 years (including the current year) and the following 10 years (excluding the current year), respectively (Nowacki and Abrams, 1997). A GC% value greater than 25% signifies a growth release, whereas a value below -10% indicates growth suppression (Rubino and McCarthyz, 2004; Ariya et al., 2016; Amoroso and Blazina, 2020). A growth release or suppression event is recorded when more than 50% of the trees at a given sampling site concurrently exhibit either a growth release or a growth suppression in a particular year. Higher percentages reflect greater disturbance intensity. Forest resilience is represented by growth recovery (Rc) and resilience (Rs) as proposed by Lloret et al. (2011), Rc = PostDr/Dr and Rs = PostDr/PreDr. where Dr indicates the index of site tree-ring chronology in the year of climatic extreme, and PreDr and PostDr indicate the means of chronology indices during the four years before and after the disturbance events.

## 3 Results

### 3.1 Species composition of the sampling site

A total of 3669 plants were recorded across the six sites in the *Abies fanjingshanensis* community. These plants belong to 32 families, 50 genera, and 72 species. The canopy layer comprises 27 species from 15 families and 20 genera, the subcanopy layer includes 48 species from 23 families and 35 genera, and the herbaceous layer consists of 19 species from 8 families and 14 genera. The number of plants in each layer and the species counts per site are summarized in **Table 1**. The importance value of *Abies fanjingshanensis* in the tree layer (9.07%) is lower than that of *Tsuga chinensis* (11.98%), *Enkianthus chinensis* (11.83%), *Rhododendron oligocarpum* (10.96%), and *Litsea rubescens* (9.79%), and it ranks only 11th in the shrub layer (**Table 2**).

**Table 1:** Number of plants and species in the canopy, subcanopy, and herbaceous layers at each site.

No. of sites	Canopy layer				Subcanopy layer		Herbaceous layer	
	Number of plants	Number of species	Mean DBH (cm)	Canopy cover	Number of plants	Number of species	Number of plants	Number of species
YD-1	103	16	10.07	62%	472	35	12	8
YD-2	77	14	11.30	71%	752	29	22	10
YD-3	133	14	8.97	68%	566	24	10	8
YD-4	146	14	8.44	57%	435	21	5	3
YD-5	120	13	9.46	66%	319	19	1	1
YD-6	88	16	10.76	67%	401	26	7	6
Total	667	27	9.62	63%	2945	47	57	19

**Table 2:** Top 15 species ranked by importance value and Niche Breadth in the canopy and subcanopy layers of the *Abies fanjingshanensis* community.

Layer	No.	Species (Number)	Important value (IV) %	Niche breadth (*B_L_*)
Canopy Layer	q1	*Tsuga chinensi*s (98)	11.98	0.948
	q2	*Enkianthus chinensis* (106)	11.83	0.789
	q3	*Rhododendron oligocarpum* (96)	10.96	0.870
	q4	*Litsea rubescens* (86)	9.79	0.822
	q5	*Abies fanjingshanensis* (51)	9.07	0.872
	q6	*Acer flabellatum* (66)	8.98	0.979
	q7	*Rhododendron argyrophyllum* (45)	7.55	0.741
	q8	*Prunus serrula* (36)	6.27	0.922
	q9	*Ilex fargesii* (6)	4.06	0.960
	q10	*Eurya nitida* (16)	3.58	0.975
	q11	*Sorbus keissleri* (8)	2.70	0.751
	q12	*Betula insignis* (14)	1.71	0.000
	q13	*Hydrangea longipes* (5)	1.55	0.356
	q14	*Ilex bioritsensis* (6)	1.42	0.179
	q15	*Abelia uniflora* (3)	1.37	0.396
Subcanopy Layer	g1	*Rhododendron oligocarpum* (616)	18.74	0.780
	g2	*Eurya nitida* (567)	12.69	0.905
	g3	*Enkianthus chinensis* (249)	7.24	0.908
	g4	*Acer flabellatum* (161)	5.83	0.875
	g5	*Rhododendron argyrophyllum* (247)	5.79	0.825
	g6	*Rubus pentagonus* (187)	4.26	0.611
	g7	*Tsuga chinensis* (103)	3.83	0.978
	g8	*Hydrangea longipes* (123)	3.66	0.788
	g9	*Abelia uniflora* (74)	3.43	0.575
	g10	*Litsea rubescens* (84)	3.19	0.924
	g11	*Abies fanjingshanensis* (85)	3.07	0.839
	g12	*Ilex fargesii* (91)	2.66	0.595
	g13	*Berberis xanthoclada* (55)	2.45	0.905
	g14	*Yushania complanate* (63)	2.35	0.654
	g15	*Mahonia polyodonta* (34)	2.07	0.904

### 3.2 Tree-ring series

**Figure 2:**
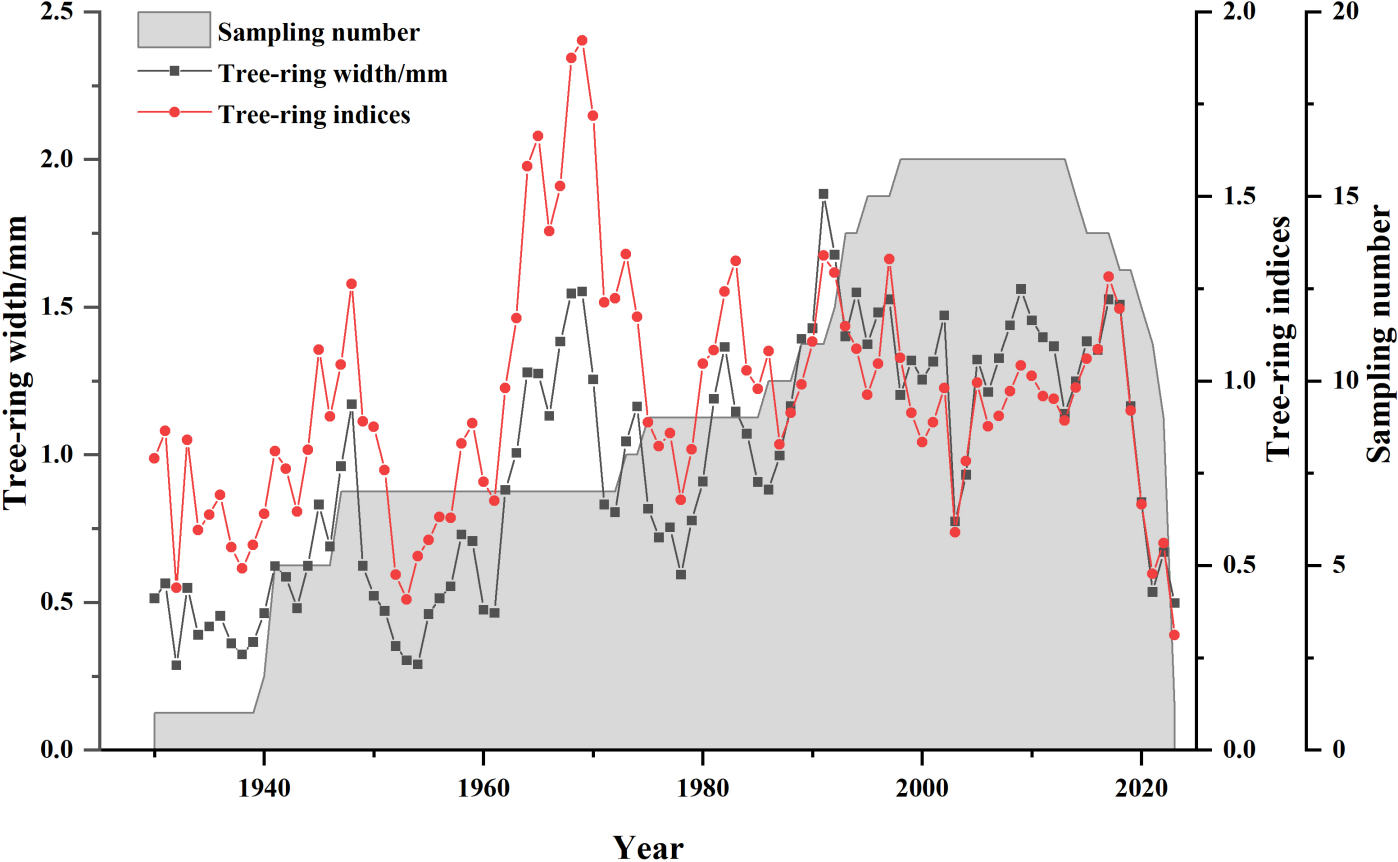
Raw tree-ring width chronology (black line) and ring-width index chronology (red line) of the 16 *Abies fanjingshanensis* samples at Mount Fanjing.

The mean annual radial increment of *Abies fanjingshanensis* was 1.439 mm/year. The average age of the sampled trees was 54 years, with a maximum age of 93 years (**Figure 2**). Cross−dating analysis revealed an interseries correlation coefficient of 0.447 and a mean sensitivity of 0.236. The signal-to-noise ratio (SNR) was 5.150, and the expressed population signal (EPS) was 0.837, exceeding the commonly accepted threshold of 0.85 when the number of trees in the sample was greater than five. These statistics indicate that sampled trees are highly representative of the study forest.

### 3.3 Limiting factors of tree growth

**Figure 3:**
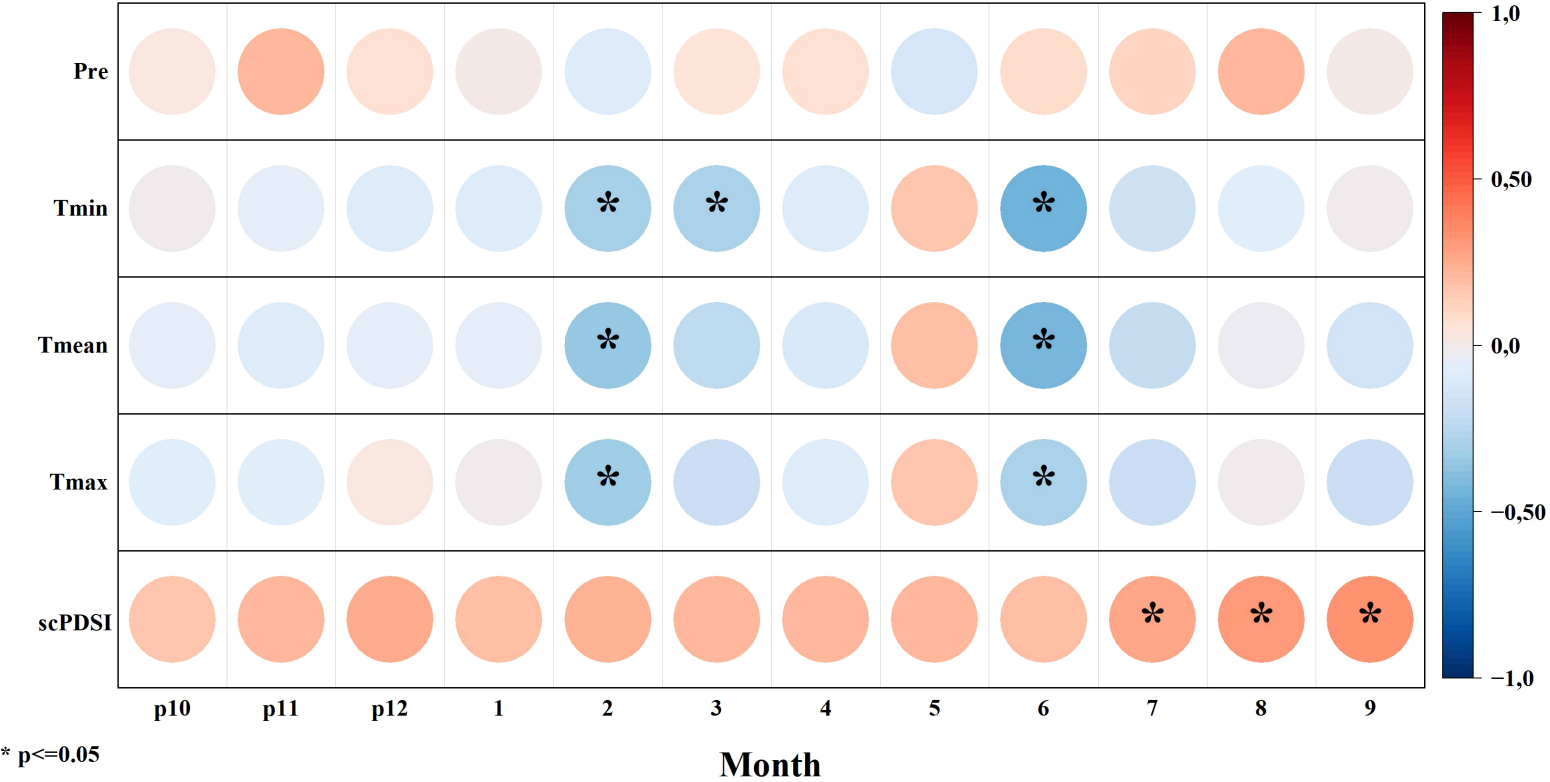
Correlation coefficients between the growth of *Abies fanjingshanensis* and climatic factors. p represents the month of the previous year; Pre represents monthly total precipitation; Tmin, Tmean, and Tmax denote monthly minimum, mean, and maximum temperature, respectively; and scPDSI refers to the self-calibrating Palmer Drought Severity Index; * refers a significant correlation between tree growth and climatic factors (*p* < 0.05).

**Figure 4:**
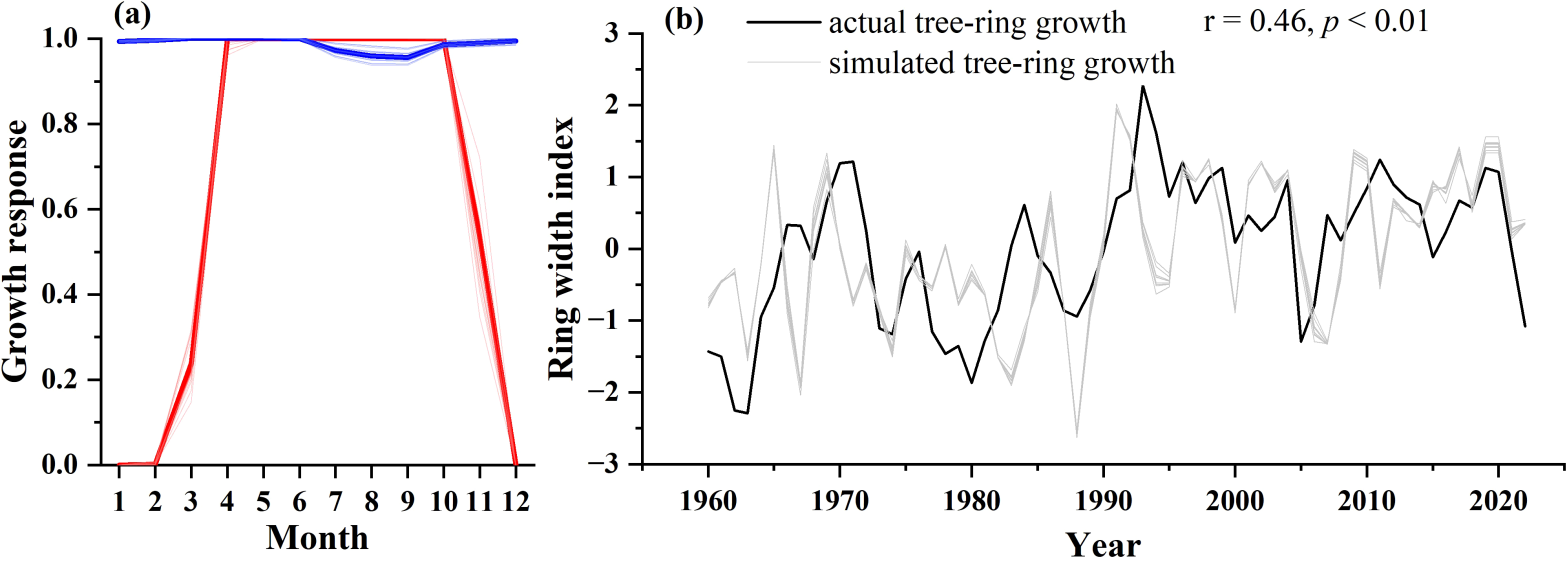
Simulated monthly growth response curves and simulated tree-ring growth of *Abies fanjingshanensis*. **(a)** The red curve represents the growth rate curve limited by monthly mean temperature, while the blue curve represents the growth rate curve limited by soil moisture. In each month, the lower value indicates which specific limiting factor is constraining tree growth. **(b)** Black line represents the actual tree-ring growth of *Abies fanjingshanensis* and gray lines represent the simulated tree-ring growth.

No significant correlation was detected between the radial growth of *Abies fanjingshanensis* and precipitation. However, growth was significantly negatively correlated with temperature in February and June (*p* < 0.05) and significantly positively correlated with scPDSI from July to September (*p* < 0.05) (**Figure 3**). Based on the VS-Lite model, growth of *Abies fanjingshanensis* occurs when the temperature rises above 9 °C in March (T1, lower temperature threshold for tree growth), and temperature ceases to be a limiting factor once it exceeds 13.5 °C (upper temperature threshold beyond which temperature no longer limits tree growth). Radial growth is primarily limited by soil moisture during the period from July to October (**Figure 4**). A significant correlation was found between the observed and simulated tree−ring growth (r = 0.46, *p* < 0.01).

### 3.4 Disturbance history of *Abies fanjingshanensis* forest

**Figure 5:**
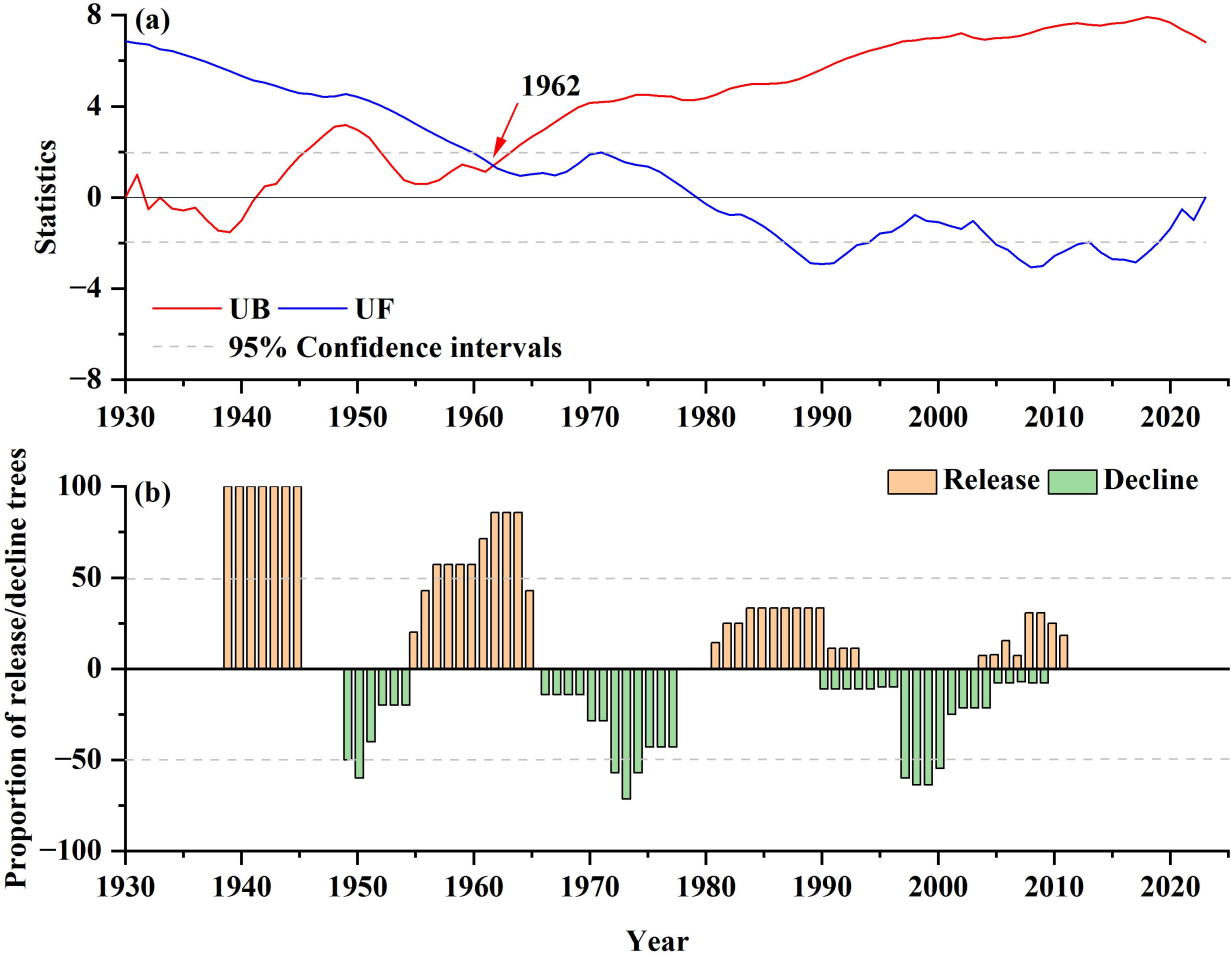
MK test results and historical growth release or suppression events for *Abies fanjingshanensis*. **(a)** MK test for *Abies fanjingshanensis*, where UF and UB are statistics used in the MK test to quantify the direction and strength of the trend, which represent the ordered series statistic and backward series statistic, respectively. **(b)** Historical growth release or suppression events identified for *Abies fanjingshanensis*.

The radial growth of *Abies fanjingshanensis* exhibited a significant increasing trend during the 1960s. A growth release event occurred between 1962 and 1965, during which 80% of the sampled trees experienced release. Growth suppression events were detected in the early 1970s and around the turn of the 21st century, with suppressed trees accounting for over 50% of the total in both periods. Over the most recent decade, *Abies fanjingshanensis* has shown a pronounced declining trend (**Figures 2**, **5**).

## 4 Discussion

### 4.1 Growth limiting factors of *Abies fanjingshanensis*

Previous investigations of vegetation-climate relationships in the Fanjingshan region have mainly focused on paleoclimate reconstruction based on pollen records (Gao et al., 2019), ecological stoichiometric characteristics of forest soils (Xu et al., 2022), and elevational distribution patterns of soil properties (Luo et al., 2024). In contrast, quantitative research on the relationship between radial tree growth and climatic factors in this region remains extremely limited. Studies on other fir species of the same genus, such as *Abies yuanbaoshanensis* in areas adjacent to Guizhou and *Abies georgei* in Southwest China, have identified summer high temperature as the dominant growth-limiting factor, with moisture conditions exerting only a weak influence (Huang, 1998; Yin et al., 2018; Sun et al., 2021; Yuan et al., 2023). In habitats with steeper slopes and poor soil water-holding capacity, water availability often becomes the more critical limiting factor. Given its unique habitat such as steep slopes, proximity to ridges, high humidity coupled with rapid soil drainage (Li et al., 2011; Lin and Mou, 2021), *Abies fanjingshanensis* is likely to exhibit climatic responses distinct from those of other fir species, with water availability emerging as a predominant limiting factor.

Our results reveal that the growth of *Abies fanjingshanensis* exhibits a significant negative correlation with June temperature and a significant positive correlation with July-September soil moisture (scPDSI), but shows no significant response to total monthly precipitation. This finding aligns with previous work on soil moisture dynamics in the region. Although annual precipitation exceeds 1,100 mm and relative humidity reaches 80%, the steep slopes (50°-60°) promote rapid runoff, resulting in inherently low soil water-holding capacity (Li et al., 2011; Lin and Mou, 2021). Consequently, growth limitation during the mid-growing season is driven by water availability rather than by total precipitation amounts.

From a physiological perspective, high temperatures inhibit *Abies fanjingshanensis* growth through three principal pathways. First, high temperatures increase vapor pressure deficit (VPD), which triggers stomatal closure to reduce water loss; this, in turn, restricts CO_2_ diffusion and lowers net photosynthetic rates (McDowell and Allen, 2015; McDowell et al., 2020). Second, prolonged exposure to high temperatures can induce carbon starvation, where respiratory carbon losses exceed photosynthetic gains, thereby depleting non-structural carbohydrate reserves (Adams et al., 2013). Third, elevated temperatures heighten the risk of xylem embolism, disrupting the integrity of the water transport system and ultimately causing hydraulic failure (Anderegg et al., 2013).

Mechanistically, June is a critical window for early xylem formation in *Abies fanjingshanensis*. High temperature stress during this period directly disrupts cell division and wall thickening, thereby significantly reducing radial increment. Conversely, July-September encompasses the mid-to-late growing season, when soil water deficits restrict carbon fixation and xylem cell expansion, further limiting radial growth. Taken together, these findings establish growing-season water availability as the dominant constraint on *Abies fanjingshanensis* productivity, providing a mechanistic, climate-informed explanation for the species’ vulnerability.

### 4.2 Historical disturbance events

Application of the percent growth change (GC%) method and the Mann-Kendall trend test revealed three significant disturbance events in the *Abies fanjingshanensis* population. These were a growth release during the early 1960s, a growth suppression in the early 1970s, and a further suppression near the close of the 20th century. Moreover, the past decade has witnessed a sustained, unrecovered growth decline accompanied by individual mortality.

The growth release event of the early 1960s coincided with a severe nationwide drought (1959-1961). Although direct evidence of canopy mortality in competing species is lacking, extreme drought has been shown to cause differential mortality across tree species in other forest ecosystems (Allen et al., 2015; McDowell et al., 2020). It is plausible that the drought reduced the vigor or survival of some competing broadleaved or coniferous trees in the Fanjingshan forest, thereby temporarily lowering competition. The surviving *Abies fanjingshanensis* individuals would have gained increased access to light, water, and nutrients, triggering a rapid growth release (Nowacki and Abrams, 1997). Post−drought growth rebound has been observed in other fir species following canopy disturbance (Linares et al., 2010; Trouvé et al., 2017).

The growth suppression events detected in the early 1970s and at the end of the 20th century may be attributed to two interrelated factors, intensified competition driven by rising stand density, and the cumulative legacy of regional drought years. The canopy opening triggered by the early-1960s growth release initially permitted surviving trees to grow rapidly with ample resources. As the canopy gradually closed and stand density increased, competition for light, water, and nutrients among individuals re-emerged and intensified (Linares et al., 2010; Trouvé et al., 2017; Cao et al., 2021). Within high-density stands, *Abies fanjingshanensis* is a relatively poor competitor and thus faces prolonged resource scarcity, which manifests as persistent radial growth inhibition (**Table 2**). This competitive disadvantage may limit its ability to acquire resources, particularly during periods of water stress. Studies have shown that competition intensity can modulate tree growth responses to climate, with dominant species often having priority access to soil moisture and light resources (Canham et al., 2006). Therefore, the sensitivity of *Abies fanjingshanensis* to drought may partly stem from its marginal niche position within the community. Moreover, competitive stress lowers the threshold of tolerance to additional stressors such as drought, predisposing trees to growth decline even when environmental pressures are only moderate.

**Figure 6:**
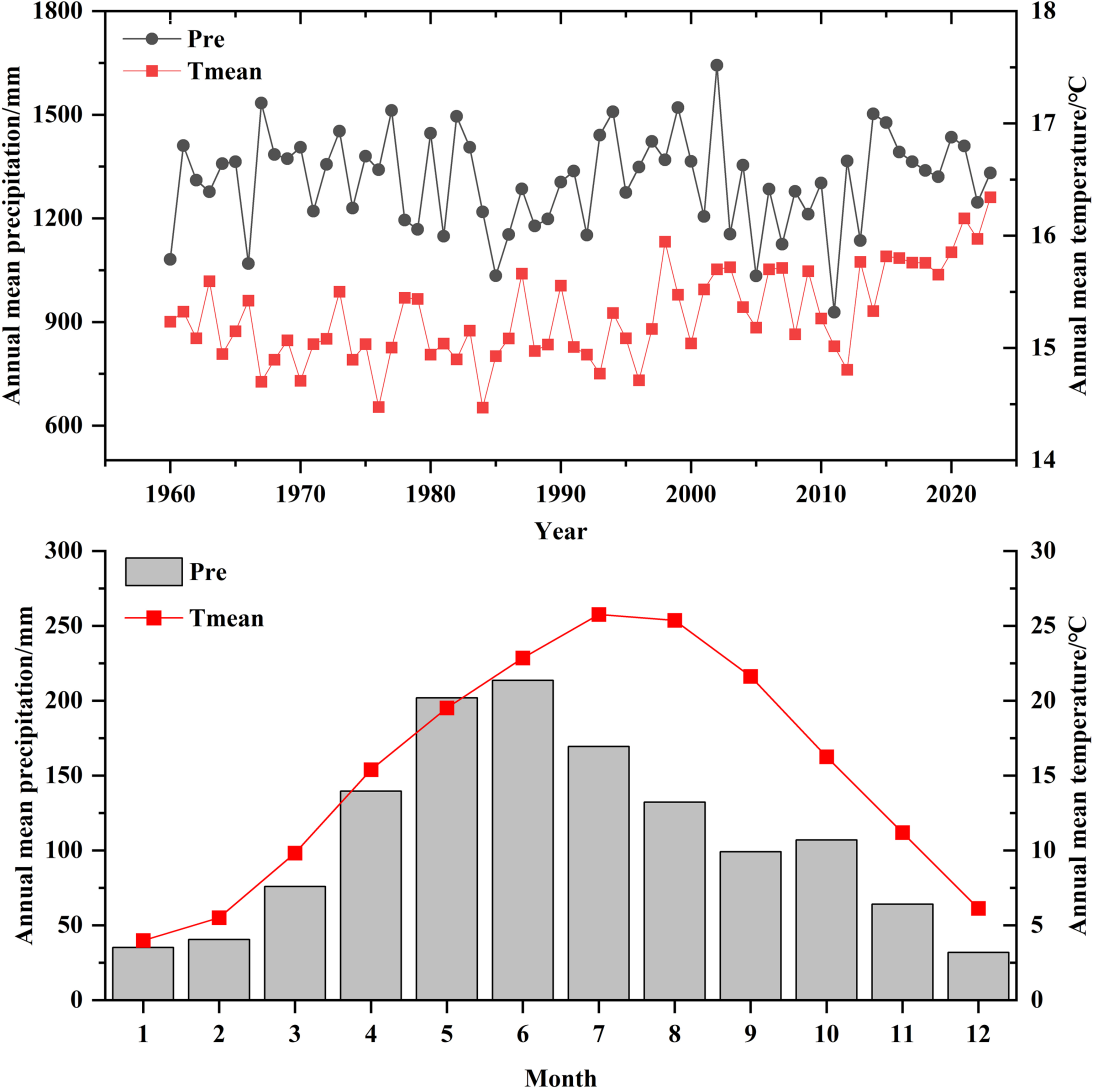
Meteorological diagram and annual variations of temperature and precipitation since 1960 at the Mount Fanjing as recorded from the grid data.

Furthermore, while a single moderate drought may not directly induce pronounced growth suppression, repeated or prolonged drought over consecutive years can progressively deplete carbon reserves and impair hydraulic transport capacity through cumulative effects. Notably, the suppression events of the early 1970s and the late 20th century were each preceded by periods of drought years of varying severity (**Figure 6**). This accumulated stress hinders the capacity of trees to restore normal physiological function in subsequent growing seasons, ultimately manifesting as a reduction in radial growth (Bigler et al., 2006; Gao et al., 2018).

**Figure 7:**
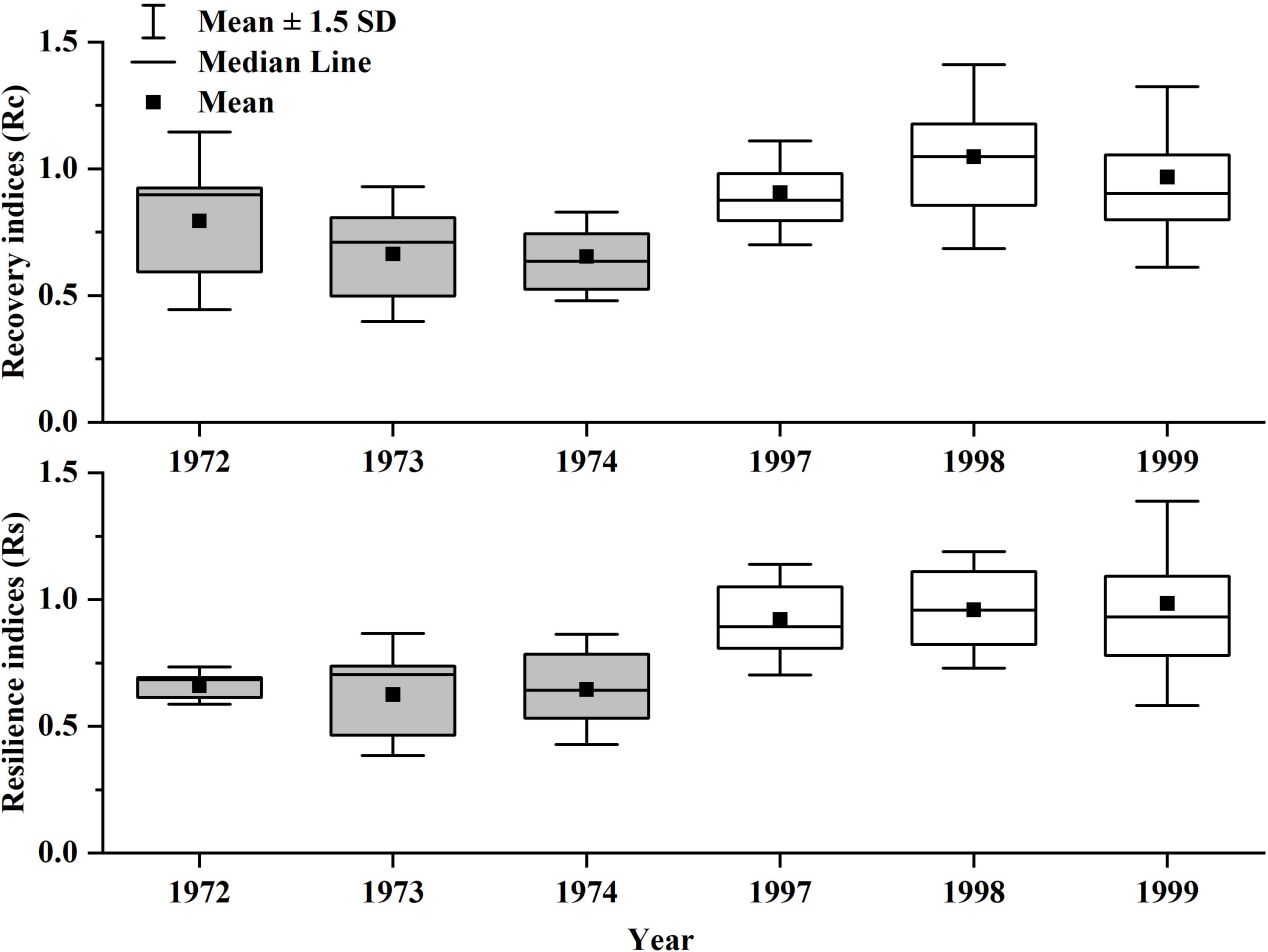
Forest recovery and resilience after disturbance events.

The recent decline differs from historical disturbances in its persistence, broad extent, and irreversibility. Since the 2010s, the Fanjingshan region has experienced a continued rise in annual mean temperature alongside declining summer precipitation (**Figure 6**), a warming−drying trend that may exceed the physiological tolerance limits of the species (Allen et al., 2015; McDowell et al., 2020). Enhanced evapotranspiration has induced long−term soil water deficits, while increasingly frequent extreme heat events can trigger irreversible xylem hydraulic failure (Anderegg et al., 2013). Although the population showed post−drought recovery in the 1960s, a release of comparable magnitude did not occur in later periods. Under current climatic conditions characterized by frequent and intense droughts, despite an increase in resilience (**Figure 7**), the replenishment of non−structural carbohydrates and the repair of hydraulic function are severely constrained, ultimately driving sustained growth decline and individual mortality (Adams et al., 2013; McDowell and Allen, 2015).

## 5 Conclusions

Both high summer temperature and water deficit constrain the growth of *Abies fanjingshanensis*, with moisture availability being the dominant limiting factor during the growing season. Within its community, the species occupies a subordinate competitive position and exhibits limited capacity for resource acquisition, traits that collectively heighten its sensitivity to drought. Although the population has shown some resilience following extreme disturbances, the persistent warming and declining precipitation observed over the past decade have driven substantial growth decline and individual mortality, suggesting a possible exceedance of its tolerance limits. These findings reveal the species’ sensitivity to water availability and its past disturbance responses. We suggest that these factors should be considered in future conservation planning. Our results point to critical priorities for further research and long-term monitoring, including refined assessments of habitat suitability, quantitative evaluation of competitive pressure, and *ex situ* conservation. Such efforts will be essential to better understand the ecological resilience and long-term viability of *Abies fanjingshanensis* populations under ongoing climate change.

## Data Availability

The original contributions presented in the study are included in the article/supplementary material. Further inquiries can be directed to the corresponding author.
